# Retraction: Novel Insights into the Synergistic Interaction of a Thioredoxin Reductase Inhibitor and TRAIL: The Activation of the ASK1-ERK-Sp1 Pathway

**DOI:** 10.1371/journal.pone.0231103

**Published:** 2020-03-25

**Authors:** The PLOS ONE Editors

After publication of this article [1] concerns were raised about the following results reported in Figs 1, 2, and 4:

- In Fig 1E, the dot plots for TRAIL, DTCD, and DTCD+TRAIL+z-VAD include several regions of similarity.
- In Fig 1F, there are similar clusters of stained cells in the Control and DTCD+TRAIL+zVAD panels. The areas of similarity abut areas within each image that otherwise differ across panels.
- In Fig 2D, in the A2780 cell Caspase 3 panel there appears to be a white rectangle under the upper bands in lanes 4–8. The authors commented that this rectangle was added and used for panel annotation during figure preparation; the original raw image from this experiment is available from the corresponding author.
- In Fig 4C, in the SKOV3+DTCD Cytosol β-actin panel, lanes 1, 2 appear similar to lanes 3, 4. The authors noted that these lanes were duplicated in error during figure preparation, and they provided blot image data to support these results.

The authors acknowledged errors in preparing Fig 1E. After reviewing the laboratory records, they stated that all panels in Fig 1E except for the DTCD+TRAIL panel were generated using the wrong raw data files. They further commented that the similar panels noted above appear to have been derived from the same raw flow cytometry (.fcs) file using different gating parameters, due to errors in file labeling and/or data analysis. The authors have since re-analyzed data from this experiment using the same quadrant and gating parameters; raw data files and results of the post-publication analyses are available from the corresponding author.

With regard to Fig 1F, the authors commented that images from different files may have been combined during image processing and/or figure preparation. The raw data underlying Fig 1F are no longer available and as such the *PLOS ONE* Editors cannot clarify how the published figure panels relate to the original experimental output. The authors provided results from replication experiments to support these results, but they did not clarify the concerns regarding the published figure.

TL apologized for the issues with Fig 1 and requested retraction, yet noted that they stand by the article’s conclusions.

In addition, the authors clarified that in Fig 2C, the rectangles for Cytosol fraction, COX IV, and for Mitochondria fraction, β-actin, are intentionally devoid of image data; the β-actin and COX IV loading controls were run only for the Cytosol and Mitochondria fractions, respectively.

The original data for other results reported in the article are available from the corresponding author.

Upon consultation with a member of *PLOS ONE*’s Editorial Board, concerns remain about the nature of the issues raised for Figs 1 and 4.

In light of these remaining concerns about the reliability of the published results, the *PLOS ONE* Editors retract this publication.

TL and YC agree with retraction. ZD, GL, JL, and JS either could not be reached or did not reply.

## References

[1] Lin T, Chen Y, Ding Z, Luo G, Liu J, Shen J (2013) Novel Insights into the Synergistic Interaction of a Thioredoxin Reductase Inhibitor and TRAIL: The Activation of the ASK1-ERK-Sp1 Pathway. PLoS ONE 8(5): e63966 10.1371/journal.pone.0063966 23696862 PMC3655947

